# Hemophagocytic Lymphohistiocytosis (HLH) in an Elderly Male With Epstein-Barr Virus (EBV) Viremia

**DOI:** 10.7759/cureus.64336

**Published:** 2024-07-11

**Authors:** Mona Ghias, Hugo Carducci, Leslie-Joy Romero, Asif Haris, Lindsay Sunzeri

**Affiliations:** 1 Internal Medicine, West Virginia University, Morgantown, USA; 2 Internal Medicine, West Virginia University School of Medicine, Morgantown, USA; 3 Pediatric Hospital Medicine and Internal Medicine, West Virginia University School of Medicine, Morgantown, USA

**Keywords:** acute hepatic failure, ebv positive elderly, ebv positive lymphoproliferative disorder, hemophagocytic lymphohistiocytosis (hlh), hlh

## Abstract

This is a case of a 75-year-old male with a complicated past medical history who presented initially with weakness, fevers, exertional dyspnea, cough, and confusion. His initial workup revealed elevated aspartate transaminase (AST), alanine transaminase (ALT), bilirubin, and D-dimer. Right upper quadrant (RUQ) ultrasound revealed a partially contracted gallbladder with gallstones, so he underwent laparoscopic cholecystectomy. Due to worsening hyperbilirubinemia and anemia, he later underwent a liver biopsy which showed Epstein-Barr virus (EBV)-positive lymphoid infiltration. He developed anemia, thrombocytopenia, and low fibrinogen. He met the criteria for hemophagocytic lymphohistiocytosis (HLH) with 6/8 HLH-2004 criteria and an H score of 230 with a 96-98% probability of HLH. The patient was promptly treated with steroids, rituximab, and etoposide; however, the patient’s health continued to deteriorate, and he expired. This case highlights the challenges of early diagnosis of HLH in the elderly patient population due to large differentials, confounding comorbidities, and the rarity of the diagnosis in this age range.

## Introduction

Hemophagocytic lymphohistiocytosis (HLH) is a life-threatening inflammatory syndrome that is characterized by uncontrolled immune activation and hemophagocytosis (destruction of red blood cells by macrophages) [1]. HLH is seen most commonly in neonates or young adults, and can either be inherited, secondary to infections (Epstein-Barr virus (EBV) most commonly), or secondary to malignancy [2]. The disease can quickly lead to multi-organ failure, commonly involving organs such as bone marrow, spleen, kidney, lungs, and liver, which can mimic sepsis, making a quick diagnosis and early intervention challenging [3]. HLH should be suspected when any of the characteristic signs and symptoms of HLH are present which include fever, splenomegaly, blood cytopenia, hepatitis and/or hepatomegaly, coagulopathy, and central nervous system disturbances [4]. Patients with hepatic involvement can progress to acute liver failure, which necessitates a liver transplant emergently in some cases [5]. As HLH can mimic many conditions, including liver failure and septic shock, the diagnosis may easily be missed. Mortality can be as high as 50% if not treated early, making it extremely important to diagnose early and initiate potentially life-saving therapy [3,4]. Here we present a case of a 75-year-old male with a complex medical history including coronary artery disease and hemochromatosis who was initially treated for suspected cholangitis and cholecystitis with laparoscopic cholecystectomy and antibiotics, but continued to deteriorate and developed blood cytopenia, coagulopathy, hepatitis, and altered mental status. He was diagnosed with HLH from EBV infection. Despite the swift initiation of treatment, the patient's condition deteriorated, and he expired.

## Case presentation

The patient is a 75-year-old male with a past medical history of coronary artery disease with previous coronary artery bypass grafting (CABG) and hypertension initially presented to his primary care provider (PCP) with generalized weakness, fevers, exertional dyspnea, cough, and confusion. After failed treatment with oral steroids and azithromycin for suspected pneumonia, his PCP did further workup where he found elevated alanine transaminase (ALT), aspartate transaminase (AST), alkaline phosphatase (ALP), and total and direct bilirubin (Table 1). The patient also had intermittent fevers. He was sent to the hospital and was admitted for further workup. Initial investigation showed negative antineutrophilic antibody (ANA), HIV, and hepatitis A, B, and C serologies. CT imaging of the abdomen and chest showed splenomegaly and diffuse lymphadenopathy in the chest and abdomen. Right upper quadrant (RUQ) ultrasound at that time showed a contracted gallbladder with gallstones. The patient underwent laparoscopic cholecystectomy for suspected cholecystitis. A cholangiogram ruled out an obstruction in the bile duct. Postoperatively, the patient still had worsening liver function tests as well as low platelets and hemoglobin (Table 1).

**Table 1:** Test results during hospital course AST: aspartate aminotransferase; ALT: alanine aminotransferase; ALP: alkaline phosphatase; LDH: lactate dehydrogenase; EBV: Epstein-Barr virus; PCR: polymerase chain reaction

Test	Initial results on presentation	Results near end of hospital course	Reference range
Hemoglobin	12.6 g/dl	9.1 g/dl	13.5-17.5 g/dl
Platelet	131 x 10^3^/ul	37 x 10^3^/ul	150-400 x 10^3^/Ul
AST	160 U/l	868 U/l	8-33 U/l
ALT	340 U/L	362 U/l	4-36 U/l
ALP	181 U/L	213 U/l	45-115 U/L
Total bilirubin	4 mg/dL	19.5 mg/dL	0-0.3 mg/dL
Conjugated bilirubin	3.5 mg/dl	13.6 mg/dl	0.1-0.4 mg/dl
LDH	547 U/L	871 U/l	125-220 U/L
Ferritin	17,892 ng/ml	7276 ng/ml	24-336 ng/ml
Fibrinogen	130 mg/dl	141 mg/dl	200-400 mg/dl
EBV quantitative PCR	966,440 copies	87,971 copies	<0.9 U/mL
Triglycerides	415 mg/dl	698 mg/dl	<150 mg/dl

Hematology/oncology and gastroenterology were on board for intra-abdominal and mediastinal lymphadenopathy. The patient underwent endoscopic ultrasound with liver and lymph node biopsy.

**Figure 1:**
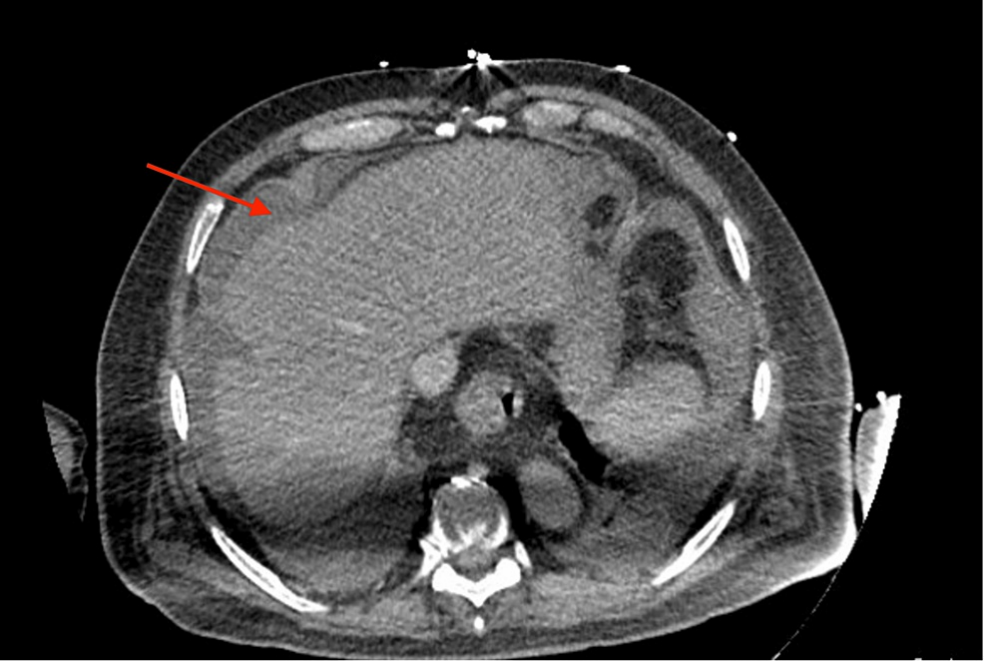
Computed tomography scan of sub-capsular hematoma

Post-biopsy, the patient developed massive bleeding in the liver (Figure 1) leading to hemoperitoneum and hemorrhagic shock. The patient was emergently taken for an exploratory laparotomy and liver packing. Subsequently, the patient required interventional radiology (IR) embolization of the hepatic artery to control the bleeding. The patient developed worsening anemia, thrombocytopenia, and low fibrinogen. He required multiple cryoprecipitate transfusions and vasopressor support. Iron studies revealed a ferritin of 17,892 ng/ml.

Results from liver and lymph node biopsies demonstrated dense/atypical EBV-positive lymphoid infiltrate with a decreased CD4:CD8 ratio. EBV quantitative PCR revealed a high viral load (Table 1). Flow cytometry was unremarkable. The patient had elevated ferritin, elevated triglycerides, thrombocytopenia, anemia, low fibrinogen, and worsening hepatic function panel (Table 1). Even though biopsies were negative for histiocytosis or hemophagocytosis, the patient was diagnosed with HLH based on a high HLH score of 230 which had a probability of 96-98% for HLH [5,6]. The patient was promptly initiated on etoposide, rituximab, and dexamethasone but he continued to progress into multiorgan failure and subsequently expired.

## Discussion

HLH is a rare disease caused by a dysregulated immune response from either an inherited genetic mutation or from a secondary source: an infection, malignancy, or autoimmune disease [6]. Accurate epidemiological data is difficult to obtain on HLH due to imprecise diagnostic criteria and multiple confounding illnesses during the diagnosis [6]. One study from the Swedish national registry concluded that primary HLH has an incidence of roughly 1.5 per million [7]; however, the incidence and prevalence are not entirely established due to the rarity of the condition and the difficulty in establishing a diagnosis [8,9].

The presenting signs and symptoms of HLH including fever, hepatosplenomegaly, lymphadenopathy, neurologic involvement, etc. are nonspecific and overlap with multiple other inflammatory and infectious diseases which makes it difficult to diagnose [8]. Other viral infections associated with HLH are cytomegalovirus (CMV), adenovirus, and influenza [9]. Diagnostic criteria for HLH include but are not limited to fever >38.5, splenomegaly, cytopenias, and ferritin >500ng/ml [9]. The most common laboratory abnormalities according to HLH-2004 guidelines are cytopenia, hypertriglyceridemia, hypofibrinogenemia, elevated ferritin, elevated liver enzymes, hyperbilirubinemia, and elevated CRP [9]. Histiocytosis and hemophagocytosis add significant value to the diagnosis if seen on a biopsy of the lymph nodes, spleen, or bone marrow; however, these are not always present during diagnosis [10].

Primary HLH is extremely rare in the elderly as evidenced by only a few cases in the literature [11]. As in our patient, infections are one of the most common triggers of HLH in the elderly population [12]. Given the age-related decline in immunity in the elderly, EBV reactivation is very common which can then lead to HLH [13]. It is important to consider the probability of HLH in an elderly patient presenting with fever, hepatosplenomegaly, cytopenia, elevated liver enzymes and bilirubin, and elevated ferritin, etc. [8]. Treatment of HLH includes but is not limited to dexamethasone, etoposide, rituximab, and antiretrovirals. As in our patient, multiorgan failure is one of the leading causes of mortality in HLH which happens mostly due to excessive cytokine release from a hyperactive immune system [14].

## Conclusions

This case highlights the complexities of diagnosing HLH in an elderly patient with multiple co-morbidities. HLH should be in the differential diagnosis of elderly patients with a constellation of clinical findings and laboratory results including fever, cytopenia, coagulopathy, elevated ferritin, and lymphadenopathy, especially when the patient experiences clinical deterioration despite treatment for initial diagnoses. Early recognition and prompt treatment initiation are critical for improving the prognosis of HLH in this vulnerable patient population with complex medical histories. It is important to maintain a broad differential diagnosis and consider HLH even in the most atypical of presentations.
